# Peptide Receptor Radionuclide Therapy in Patients with Advanced, Recurrent or Progressive Meningioma: An Updated Systematic Review and Meta-Analysis

**DOI:** 10.3390/cancers17122039

**Published:** 2025-06-18

**Authors:** Barbara Muoio, Cesare Michele Iacovitti, Davide Giovanni Bosetti, Maddalena Sansovini, Marco Cuzzocrea, Gaetano Paone, Giorgio Treglia

**Affiliations:** 1 Division of Medical Oncology, Oncology Institute of Southern Switzerland, Ente Ospedaliero Cantonale, 6500 Bellinzona, Switzerland; barbara.muoio@eoc.ch; 2 Faculty of Biomedical sciences, Università della Svizzera italiana, 6900 Lugano, Switzerland; gaetano.paone@eoc.ch; 3 Division of Nuclear Medicine, Imaging Institute of Southern Switzerland, Ente Ospedaliero Cantonale, 6500 Bellinzona, Switzerland; cesaremichele.iacovitti@eoc.ch (C.M.I.); marco.cuzzocrea@eoc.ch (M.C.); 4 Division of Radiation Oncology, Oncology Institute of Southern Switzerland, Ente Ospedaliero Cantonale, 6500 Bellinzona, Switzerland; davidegiovanni.bosetti@eoc.ch; 5 Nuclear Medicine and Radiometabolic Units, IRCCS Istituto Romagnolo per lo Studio dei Tumori “Dino Amadori”, 47014 Meldola, Italy; maddalena.sansovini@irst.emr.it; 6 Faculty of Biology and Medicine, University of Lausanne, 1015 Lausanne, Switzerland

**Keywords:** meningioma, neuro-oncology, PRRT, nuclear medicine, theragnostic, radionuclide therapy, radioligand therapy, somatostatin, meta-analysis, disease control

## Abstract

Background: Peptide receptor radionuclide therapy (PRRT) could be a therapeutic option for patients with advanced, recurrent or progressing meningiomas overexpressing somatostatin receptors. The aim of this study is to perform an updated meta-analysis to establish the disease control rate of PRRT in these patients. Methods: A comprehensive literature search of studies on PRRT in patients with advanced, recurrent or progressing meningioma was carried out. Four different databases (PubMed/MEDLINE, EMBASE, Cochrane library, Google Scholar) were screened until April 2025. Only original articles about PRRT in advanced, progressive or refractory meningiomas were selected. Case reports were excluded. Three review authors independently performed the literature search, the article selection and the data extraction. Main findings of eligible studies were summarized and a proportion meta-analysis on the disease control rate was carried out using a random-effects model. Results: In total, 18 studies (269 patients) published from 2006 to 2025 were included in the analysis. In most of the included studies, PRRT was performed using [^177^Lu]Lu-DOTATATE. The pooled disease control rate was 67.7% (95% confidence interval values: 59.6–75.7%). PRRT was well-tolerated in most patients with advanced, recurrent or progressive meningioma. Moderate statistical heterogeneity was found in the meta-analysis (I-square: 53%). Conclusions: PRRT is an effective and well-tolerated treatment in patients with advanced, progressive or recurrent meningiomas, showing a significant disease control rate (in about two-thirds of patients). Even if well-designed clinical trials are needed to corroborate these findings, evidence-based data seem to support the clinical use of PRRT for this indication.

## Simple Summary

An increasing number of studies have recently been published about peptide receptor radionuclide therapy (PRRT) as a treatment option for patients with advanced, recurrent or progressive meningioma. We have provided an updated meta-analysis on the disease control rate with PRRT in these patients. This treatment is effective and well-tolerated in most patients, showing a significant disease control rate. The updated evidence-based data seem to support the clinical use of PRRT for this indication.

## 1. Introduction

Meningiomas are the most common intracranial tumors in adult patients. While the majority are classified as benign (WHO grade 1), approximately 10–15% of cases are higher-grade tumors, which are categorized as atypical (WHO grade 2) or even malignant or anaplastic (WHO grade 3) [1].

The latest European Association of Neuro-Oncology (EANO) guidelines clearly state that observation is the primary approach for incidental asymptomatic meningiomas. Surgery remains the first-line treatment for growing or symptomatic meningiomas, while radiation therapy may serve as a complementary or even alternative option to surgery in certain cases [1]. Notably, 20–25% of meningiomas may recur after first line treatment requiring additional therapies and advanced meningiomas are tumors with a higher risk of recurrence and a more aggressive growth pattern compared to typical meningiomas [1].

Most meningiomas show high expression of somatostatin receptors (SSTR). This is the rationale for proposing nuclear medicine methods using radiolabeled SSTR-ligands for diagnosis and therapy of these tumors according to recent guidelines [2,3].

Hybrid imaging including positron emission tomography (PET) coupled with computed tomography (PET/CT) or magnetic resonance imaging (PET/MRI) using radiolabeled SSTR-ligands has been suggested in several clinical scenarios in meningiomas according to recent international guidelines [2,3]. The evidence-based indications of PET/CT or PET/MRI using SSTR-ligands in meningiomas are differential diagnosis, assessment of tumor boundaries, planning for surgery and radiation therapy, distinguishing tumor recurrence from treatment-related changes, prognostic evaluation, disease monitoring and treatment response assessment, and evaluation before SSTR-targeted therapy [2,3].

Theranostic approaches integrating SSTR-targeted imaging and radionuclide therapy have been evaluated in patients with meningioma for personalized cancer therapy [4,5,6,7,8,9,10,11].

**Figure 1:**
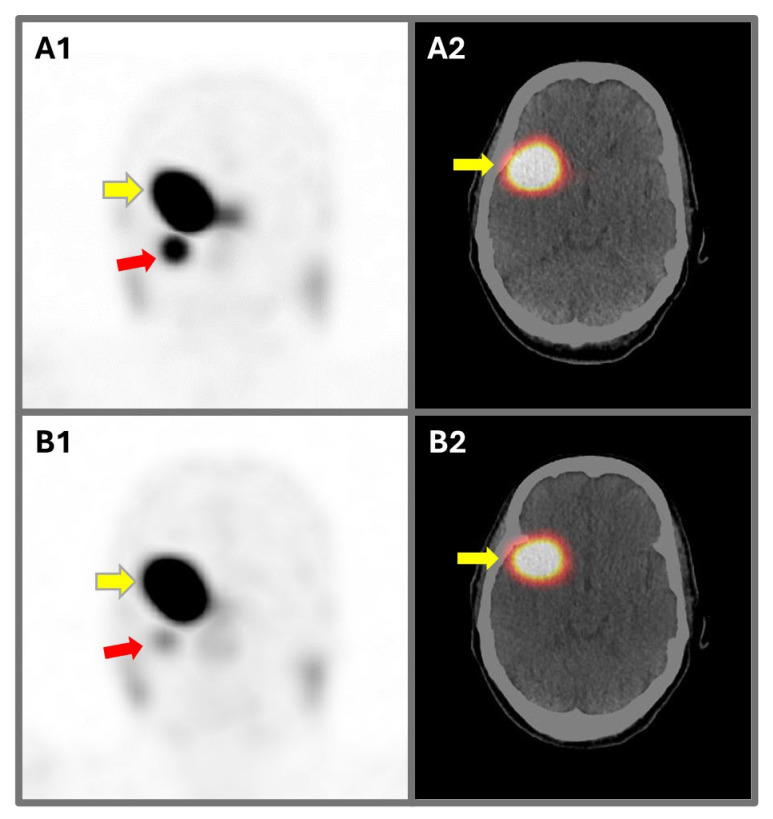
A 75-year-old female underwent peptide receptor radionuclide therapy with [^177^Lu]Lu-DOTATATE (four cycles; 29.6 GBq) for the treatment of an advanced meningioma (yellow arrows) and a concomitant paraganglioma (red arrows). Functional (A1 and B1) and hybrid images (A2 and B2) at the baseline (A1 and A2) and at the end of treatment (B1 and B2) are compared. The meningioma appeared stable, while the paraganglioma showed partial regression. Overall, disease control was obtained through the treatment.

However, peptide receptor radionuclide therapy (PRRT) using [^90^Y]- or [^177^Lu]-labeled SSTR-ligands is currently considered an investigational treatment for meningiomas, in particular in cases with advanced, progressive or refractory tumors [2,3]. An example of a patient with advanced meningioma treated with PRRT is illustrated in Figure 1.

According to a previously published meta-analysis, PRRT has been investigated in small studies, mainly including meningiomas with increased SSTR expression progressing after multiple prior treatments and when other therapy options were not applicable anymore [12]. Even if the available literature data were limited, this analysis demonstrated that PRRT was well tolerated, providing disease control in most cases [12].

As the previous meta-analysis assessing the efficacy of PRRT with SSTR-ligands in meningiomas included only six studies (published from 2006 to 2016) and 111 patients [12], and considering that several studies on this topic were published in the last 10 years [6], we would like to provide an updated evidence-based information on the efficacy of PRRT with SSTR-ligands in patients with meningioma through an updated pooled analysis of the available literature data on this topic.

## 2. Materials and Methods

### 2.1. Review Question, Working Group and Review Protocol

The review authors formulated the following review question based on selected patients, intervention and outcomes: “Which are the efficacy and safety of PRRT using radiolabeled SSTR-ligands in patients with advanced, progressive or recurrent meningiomas?”.

The review authors included one junior nuclear medicine physician (C.M.I.), four senior nuclear medicine physicians (M.S., G.T., M.C., G.P.) with experience on molecular imaging and therapy of brain tumors, one senior neuro-oncologist (B.M.) and one senior radiation oncologist (D.G.B.) with a special interest in brain tumors. Two review authors have extensive experience in systematic reviews and meta-analyses (B.M. and G.T.).

The study was carried out according to a predefined protocol [13,14]. The PRISMA checklist for this systematic review is reported as Supplemental Material.

### 2.2. Search Strategy

A thorough literature review on PRRT in meningiomas was conducted independently by three authors (C.M.I., B.M. and G.T.). Four distinct databases—PubMed/MEDLINE, Cochrane Library, Embase and Google Scholar—were systematically searched and screened until 30 April 2025. A search string was created combining several text words based on the review question: (A) “meningioma” OR “meningiomas” OR “meningioma*” AND B) “PRRT” OR “somatostatin therapy” OR “SSTR-2 therapy” OR “SSTR2 therapy” OR “radionuclide therapy” OR “radiopeptide therapy” OR “radioligand therapy” OR “octreotide therapy” OR “theragnostic*” OR “theranostic*”. No language or publication date restrictions were applied in the selected databases. To enhance the sensitivity of the literature search, the references of potentially eligible articles were also reviewed to identify additional studies.

### 2.3. Selection of Studies

The study selection was independently carried out by three review authors (B.M., C.M.I. and G.T.), who applied the predefined inclusion and exclusion criteria. Regarding inclusion, only studies or subsets of studies that investigated the efficacy and safety of PRRT with SSTR-ligands in patients with meningiomas were considered. The exclusion criteria were (a) articles outside the scope of this review (including those assessing combination therapies with PRRT and radiation therapy) or not providing information on the efficacy and safety of PRRT in meningiomas; (b) editorials, letters, reviews, comments and conference proceedings; (c) case reports related to PRRT in meningiomas.

The titles and abstracts of the records retrieved using the predefined search string in the selected databases were initially screened. After excluding ineligible records, the full texts of potentially eligible articles were downloaded and reviewed. Ultimately, studies were included in the review following a consensus meeting among three co-authors. (B.M., C.M.I. and G.T).

Articles on the efficacy and safety of PRRT included in the systematic review were eligible for the meta-analysis on the diseases control rate (selected as main clinical endpoint) whether sufficient data to calculate this outcome were available.

### 2.4. Data Extraction and Quality Assessment

Data extraction and quality assessment were carried out by two review authors (B.M. and G.T.) independently. Data extracted from the selected articles included the following: fundamental study details, patient characteristics, technical aspects of PRRT, and outcome data regarding the efficacy and safety of PRRT. The NIH quality assessment tools were used for the risk of bias/quality assessment of the selected studies [15].

### 2.5. Statistical Analysis

Disease control rate with PRRT (defined as the percentage of patients with advanced, recurrent or progressive meningioma who have achieved complete response, partial response and stable disease to PRRT) was calculated for each study included. The pooled disease control rate with PRRT was obtained through a proportion patient-based meta-analysis using a random-effects model. This statistical model considers the variability among studies. Subgroup analyses (i.e., based on the different radiopharmaceuticals used for PRRT) were performed if sufficient data were available from the included studies.

Pooled data were presented with a summary effect measure and 95% confidence interval (95%CI) values.

Statistical heterogeneity was estimated through the I^2^ statistic [13].

OpenMeta[Analyst] (version 1.0 for Windows) was used as statistical software for the meta-analysis.

## 3. Results

### 3.1. Literature Search

**Figure 2:**
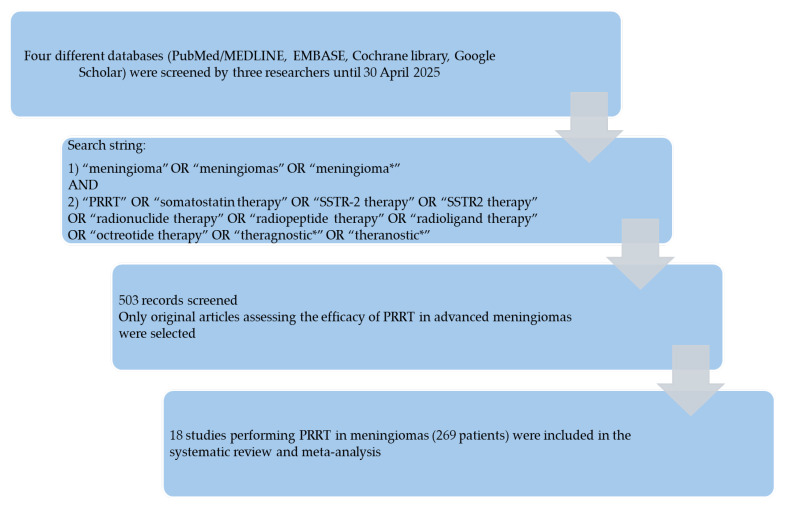
Summary of the literature search process.

Results of the systematic literature search are summarized in Figure 2: 503 records were identified using the selected databases and subsequently screened; 485 records were excluded according to the predefined exclusion criteria; and 18 studies (269 patients) were finally included in the systematic review [16,17,18,19,20,21,22,23,24,25,26,27,28,29,30,31,32,33], without additional studies found screening the reference list of the retrieved articles. Notably, two studies were excluded because they involved performing a combination of PRRT and radiation therapy [34,35], therefore the efficacy of PRRT cannot be assessed in these cases.

### 3.2. Risk of Bias Assessment

All the selected studies showed an overall moderate quality using the NIH quality assessment tool, even if randomized controlled trials were not available.

### 3.3. Qualitative Synthesis

Table 1 summarizes the main findings about original studies on PRRT in 269 patients with advanced, progressive or recurrent meningiomas published from 2006 to 2025. The included studies performed PRRT in patients with locally advanced meningiomas who were either ineligible for surgery, refractory to surgery or external beam radiotherapy, or declined surgery or external beam radiation therapy or were progressing or recurrent after multiple prior lines of treatment. Before performing PRRT, the tumors had positive expression of SSTR type 2 receptors evaluated using SSTR-directed nuclear medicine imaging [16,17,18,19,20,21,22,23,24,25,26,27,28,29,30,31,32,33].

Included studies were mainly monocentric and in most of the cases from European countries (78%). The number of patients treated with PRRT ranged from 5 to 42, median age ranged from 39 to 73 years old and male percentage from 20% to 62%. Ten studies (55.6%) included more than 10 patients. With the WHO classification of meningiomas, all the grade types were included, with a predominance of grade 1 and grade 2 tumors [16,17,18,19,20,21,22,23,24,25,26,27,28,29,30,31,32,33]. The overall percentage of meningiomas with grade 1, grade 2, grade 3 and unknown grading was 28%, 44%, 14% and 14%, respectively.

With the radiopharmaceuticals used for PRRT, most of the studies used [^177^Lu]-labeled SSTR-ligands (in particular [^177^Lu]Lu-DOTATATE) but also [^90^Y]-labeled SSTR-ligands were used in a minority of studies. The median number of PRRT cycles and the median cumulative activity significantly ranged among the studies, but four cycles and a cumulative activity of 29.6 GBq were the most frequent values for [^177^Lu]Lu-DOTATATE.

Treatment response assessment was performed through imaging methods (i.e., brain MRI, nuclear medicine and hybrid imaging with SSTR-ligands) as well as clinical evaluation and laboratory assays. MRI was used as the imaging method for the treatment response assessment in all the included studies, and nuclear medicine imaging with SSTR-ligands was used for the same purpose in about half of the included studies [16,17,18,19,20,21,22,23,24,25,26,27,28,29,30,31,32,33].

The most frequent criteria used for treatment response assessment by imaging in the included studies were the Response Assessment in Neuro-Oncology (RANO) criteria [36] (in half of included studies). Other criteria that were less frequently used were volumetric criteria [37,38], Response Evaluation Criteria In Solid Tumors 1.1 (RECIST 1.1) [39], PET Response Criteria in Solid Tumors (PERCIST) [40], Southwest Oncology Group (SWOG) criteria [41] and modified Macdonald criteria [42].

The National Cancer Institute Common Terminology Criteria for Adverse Events (CTCAE) has been used as the main classification tool for treatment-related adverse events in the included studies. With the safety, PRRT was generally well-tolerated, taking into account the information reported in the included studies. Adverse effects of PRRT are typically mild. The most common side effects of PRRT include nausea, lymphopenia and vomiting. In some studies, serious adverse events were described as transient, and the most frequently reported was grade 3 transient haematotoxicity (Table 1) [16,17,18,19,20,21,22,23,24,25,26,27,28,29,30,31,32,33].

The disease control rate ranged among the included studies from 28.6% to 87.5% [16,17,18,19,20,21,22,23,24,25,26,27,28,29,30,31,32,33]. Median progression-free survival (PFS) after PRRT ranged from 5 months to 32 months when reported, being higher for grade 1 (range: 10–47 months) compared to grade 2/3 meningiomas (range: 2–11.5 months) [16,17,18,19,20,21,22,23,24,25,26,27,28,29,30,31,32,33]. In the study of Severi et al. with the highest number of included patients with meningiomas performing PRRT (n = 42), the median PFS was 16 months, and the median overall survival was 36 months [21].

PFS rate at 12 months from the start of PRRT ranged from 34% to 87% when reported. Overall survival rate at 12 months from the start of PRRT ranged from 60% to 93% when reported. Grading was an independent prognostic factor: the risk of progression and death was higher in patients with grade 2/3 meningiomas compared to those with grade 1 meningiomas [21,23,28,29,32]. A longer survival after PRRT was found in patients with high tumor uptake of SSTR ligands than in patients with intermediate or low tumor uptake [28,29]. The study of Severi et al. reported a significantly better result of PRRT in patients with only two previous surgeries [21].

Overall, although not statistically significant, the higher PRRT efficacy observed in younger individuals, G1 meningioma, less extensive meningiomas, patients with better performance status, and asymptomatic cases suggested a potentially better treatment response in the earlier stages of the disease [21].

### 3.4. Quantitative Synthesis (Meta-Analysis)

**Figure 3:**
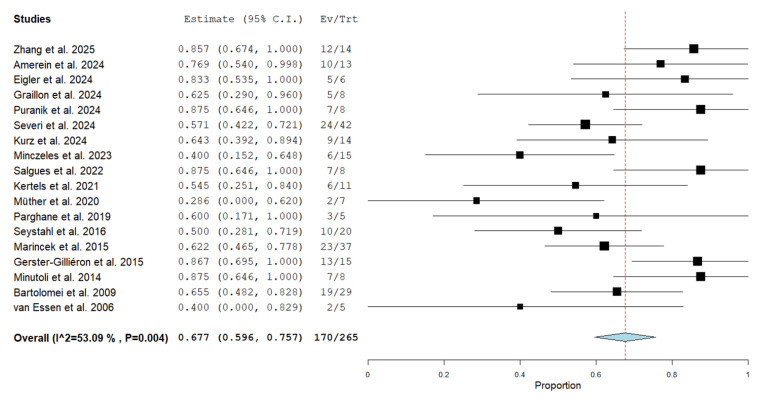
Forest plot of the meta-analysis on the disease control rate using PRRT in patients with advanced, recurrent or progressive meningiomas. The squares represent the outcome measures of each study and the horizontal lines are their 95% confidence interval values. The diamond represents the pooled value and its long axis the 95% confidence interval values [16,17,18,19,20,21,22,23,24,25,26,27,28,29,30,31,32,33].

The meta-analysis of studies using PRRT in patients with advanced, recurrent or progressive meningioma demonstrated a pooled disease control rate of 67.7% (95%CI: 59.6–75.7%). Moderate statistical heterogeneity was found (I^2^ = 53%). Figure 3 shows the forest plot of the meta-analysis.

A subgroup analysis including only studies with more than 10 patients demonstrated a similar pooled disease control rate (65.4%; 95%CI: 56.2–74.6%).

The subgroup analyses taking into account the different radiopharmaceuticals did not demonstrate significant differences among [^177^Lu]-labeled SSTR-ligands and [^90^Y]-labeled SSTR-ligands.

## 4. Discussion

Radioligand therapy including PRRT has gained considerable attention due to the recent introduction of innovative and effective theranostic agents, which have demonstrated promising therapeutic outcomes in a range of cancers [43]. Due to the increasing availability of new PET tracers for the diagnosis of brain tumors and related radiopharmaceuticals for radioligand therapy, theranostics for brain tumors and, in particular, for meningiomas is an expanding field [3,7,44,45,46]. SSTR-ligands used for PRRT are labeled with [^177^Lu]Lu or [^90^Y]Y, both β–emitters which deposit their high energy within a short range in tissues, allowing selective irradiation of the targeted tumor lesions [43].

Due to the increasing number of studies on PRRT published in recent years, this systematic review and meta-analysis provided updated information on the efficacy and safety of this therapeutic option in advanced, recurrent or progressive meningiomas. Several studies have used PRRT with SSTR-ligands in this setting [16,17,18,19,20,21,22,23,24,25,26,27,28,29,30,31,32,33], some of them with few cases included. Summary of the literature data through a systematic review and meta-analysis allowed us to offer more comprehensive data on the selected topic in comparison to individual studies.

Notably, we included 18 studies and 269 patients with advanced, recurrent or progressive meningiomas treated with PRRT in our analysis, providing a significant update and incremental value compared to the previous analysis published in 2021 and including only 6 studies and 111 patients [12]. Furthermore, the previous meta-analysis demonstrated a pooled disease control rate of 63% (95%CI: 45–81%) with PRRT. Our analysis provided a similar pooled value for this outcome measure (67.7%) but with narrower 95%CI values (59.6–75.7%), resulting in a more precise estimate and higher statistical power due to the higher number of patients included.

Overall, our updated systematic review and meta-analysis confirms that PRRT is an effective and well-tolerated treatment option in patients with advanced, recurrent or progressive meningiomas, usually already treated with surgery or radiation therapy. Obtaining a disease control rate in two-thirds of patients treated with PRRT and a good tolerability is a significant result, as these patients likely have no other therapeutic options available when treated with PRRT.

The literature data also demonstrate that several factors may predict the efficacy of PRRT in advanced, recurrent or progressive meningiomas. Young age, G1 tumor grade, less extensive tumors, better performance status, asymptomatic patients, good expressions of SSTRs and low number of previous surgeries are favorable prognostic factors suggesting a reasonably better response in the earlier stages of the disease [21].

With the safety and tolerability of PRRT in meningiomas, the most frequent serious adverse event is transient hematotoxicity, but this is not surprising considering the clinical experience with PRRT for other tumors [47]. According to the available guidelines, only pregnancy, breastfeeding (if not discontinued), severe acute concomitant illnesses and severe psychiatric disorders that cannot be managed are absolute contraindications for PRRT [3].

Notably, even if the evidence on PRRT is increasing, there is still need for adequately designed and controlled prospective multicenter trials for the effective translation of PRRT with SSTR-ligands into daily clinical practice for patients with advanced, recurrent or progressive meningiomas [2,3]. In other words, even if available studies on PRRT present encouraging results with favorable outcomes in patients with advanced, recurrent or progressive meningiomas, results from randomized clinical trials are currently missing, therefore PRRT is not yet approved for this indication by medical agencies [3].

Currently, PRRT remains an investigational treatment in patients with advanced, recurrent or progressive meningiomas, and it could be considered when other local therapy options are no longer applicable. However, until prospective and properly controlled clinical trials are available, PRRT may be offered to patients on a case-by-case basis, following a multidisciplinary evaluation [3], and results provided by our analysis clearly support this option.

Despite the widespread use of PRRT in the treatment of advanced, recurrent or progressive meningiomas, the underlying radiobiological mechanisms remain poorly understood. A deeper understanding of tumor absorbed dose in meningioma treatment could support personalized dosimetry and enable tailored therapy adjustments. Incorporating internal dosimetry data is essential to clarify the radiobiological mechanisms of molecular radiotherapy, establish dose–response relationships, and guide future clinical trials toward more personalized and optimized treatment approaches. In an effort to improve treatment efficacy, several approaches should be implemented, including the optimization of PRRT delivery protocols and the development of new radiopharmaceuticals targeting alternative somatostatin analogs or new radionuclides, particularly alpha emitters due to their superior radiobiological properties. Also, the combination of PRRT with radiosensitizers in order to improve therapeutic outcomes is warranted. Moreover, alongside the WHO classification system for meningiomas, there is a need for new molecular classification systems to enhance risk stratification and predict the response to PRRT [2,5,6,7,8,9]. Finally, combination therapies targeting different pathways are emerging, demonstrating significant potential as adjunct treatment options for meningiomas [48]. At present, medical treatments for meningiomas remain experimental due to the limited results from clinical trials. They are typically used in cases of recurrent or progressive meningiomas that cannot be addressed with surgery or radiation therapy. Despite attempts with various drugs and chemotherapy strategies, there is no definitive evidence supporting the efficacy of these therapeutic options in advanced, recurrent or progressive meningiomas [5].

According to the PRISMA statement, some limitations of our analysis should be underlined. The first limitation is the inclusion of studies with a limited number of patients in most of the cases and non-controlled trials. Second, we have found a moderate degree of statistical heterogeneity among the included studies and a significant clinical and methodological heterogeneity related to different patients’ and tumors’ characteristics and technical aspects of PRRT, respectively. However, it is unlikely that the moderate statistical heterogeneity has significantly influenced the provided pooled results on the disease control rate of PRRT.

## 5. Conclusions

Updated evidence-based data demonstrate that PRRT with SSTR-ligands is an effective and well-tolerated therapeutic option in patients with advanced, recurrent or progressive meningiomas, with a disease control rate in two-thirds of treated patients. The findings of our analysis support this therapeutic option in patients with advanced, recurrent or progressive meningiomas if other local therapy options (surgery or radiation therapy) are not applicable anymore. Results from prospective and multicentric studies are warranted for the effective translation of PRRT with SSTR-ligands into daily clinical practice for patients with advanced, recurrent or progressive meningiomas.

## Figures and Tables

**Table 1:** Main findings of studies assessing the efficacy and safety of PRRT in advanced progressive meningioma.

Authors	Year	Country	Number/Median Age/Sex Ratio (Male %) of Patients with Advanced Progressive Meningioma Treated with PRRT	WHO Classification of Meningiomas	Type of PRRT	Median Number of PRRT Cycles/Median Cumulative Activity	Patients with Disease Control After PRRT (%)	Serious Adverse Events	12-Month PFS/OS
Zhang et al. [16]	2025	Singapore and Germany	18/58 y/50%	G1 = 6; G2 = 10; G3 = 1; NR = 1	Lu-177-DOTATATE or Y-90-DOTATOC	2 cycles/10.9 GBq	12 (85.7%)*	NR	NR/NR
Amerein et al. [17]	2024	Germany	13/65 y/38%	G1 = 4; G2 = 3; G3 = 1; NR = 5	Lu-177-HA-DOTATATE	4 cycles/25.7 GBq	10 (76.9%)	transient grade 3 hematotoxicity (max 38.5%) and grade 4 hematotoxicity (7.7%)	76.9%/84.6%
Eigler et al. [18]	2024	Switzerland	6/63 y/50%	G1 = 1; G2 = 4; G3 = 1	Lu-177-DOTATOC + Lu-177-DOTA-JR11	1 + 2 or 3 cycles/7.1 + 9 GBq	5 (83.3%)	transient grade 3 hematotoxicity (50%)	NR/NR
Graillon et al. [19]	2024	France	8/72 y/62%	G1 = 1; G2 = 7	Lu-177-DOTATATE	4 cycles/29.6 GBq	5 (62.5%)	NR	75%/87.5%
Puranik et al. [20]	2024	India	8/52 y/62%	G1 = 2; G2 = 5; G3 = 1	Lu-177-DOTATATE	3 cycles/22.3 GBq	7 (87.5%)	NR	NR/NR
Severi et al. [21]	2024	Italy	42/64 y/52%	G1 = 11; G2 = 24; G3 = 4; NR = 3	Y-90-DOTATOC or Lu-177-DOTATATE	5 cycles/11.1 GBq or 4 cycles/22 GBq	24 (57.1%)	grade 3 hematotoxicity (2.4%)	NR/NR
Kurz et al. [22]	2024	USA	14/63 y/21%	G1 = 2; G2 = 11; G3 = 1	Lu-177-DOTATATE	4 cycles/29.6 GBq	9 (64.3%)	grade 3/4 events included hematotoxicity (15), electrolyte abnormalities (4), diarrhea (1), thromboembolic events (1), and cardiac arrhythmic events (2)	43%/71%
Minczeles et al. [23]	2023	Netherlands	15/52 y/47%	G1 = 3; G2 = 5; G3 = 6; NR = 1	Lu-177-DOTATATE	4 cycles/28.9 GBq	6 (40%)	grade 3 hepatic or hematological toxicity (53%) or grade 4 hepatic or haematological toxicity (7%)	NR/60%
Salgues et al. [24]	2022	France	8/72 y/62%	G2 = 8	Lu-177-DOTATATE	4 cycles/29.6 GBq	7 (87.5%)	transient grade 3 hematotoxicity (37%)	66.7%/NR
Kertels et al. [25]	2021	Germany	11/39 y/36%	G1 = 4; G2 = 6; G3 = 1	Lu-177-DOTATATE	4 cycles/29.6 GBq	6 (54.5%)	transient grade 3 hematotoxicity (18%)	45.4%/90.9%
Müther et al. [26]	2020	Germany	7/73 y/57%	G1 = 2; G2 = 5	Lu-177-DOTATATE	4 cycles/29.6 GBq	2 (28.6%)	NR	NR/NR
Parghane et al. [27]	2019	India	5/45 y/50%	G1 = 2; G2 = 4	Lu-177-DOTATATE	4 cycles/19.9 GBq	3 (60%)	NR	60%/NR
Seystahl et al. [28]	2016	Switzerland and Germany	20/43 y/45%	G1 = 5; G2 = 7; G3 = 8	Lu-177-DOTATATE or Y-90-DOTATOC	3 cycles/20.1 GBq	10 (50%)	grade 3 hematotoxicity (25%) and grade 4 hematotoxicity (5%)	NR/79%
Marincek et al. [29]	2015	Switzerland	37/61 y/26%	G1 = 5; G2 = 6; G3 = 3; NR = 23	Lu-177-DOTATOC or Y-90-DOTATOC	NR	23 (62.2%)	grade 3 hematotoxicity (8.8%)	NR/NR
Gerster-Gilliéron et al. [30]	2015	Switzerland	15/56 y/20%	G1 = 9; G2 = 2; G3 = 1; NR = 3	Y-90-DOTATOC	2 cycles/7.4 GBq/m2	13 (86.7%)	grade 3 hematotoxicity (33%)	86.7%/93.3%
Minutoli et al. [31]	2014	Italy	8/58 y/25%	G1 = 5; G2 = 3	In-111-pentetreotide	3 cycles/21 GBq	7 (87.5%)	NR	NR/NR
Bartolomei et al. [32]	2009	Italy	29/54 y/31%	G1 = 14; G2 = 9; G3 = 6	Y-90-DOTATOC	4 cycles/10 GBq	19 (65.5%)	NR	34.5%/75.8%
van Essen et al. [33]	2006	Netherlands	5/55 y/40%	G3 = 3; NR = 2	Lu-177-DOTATOC	NR	2 (40%)	NR	NR/NR

Legend: NR = not reported; OS = overall survival; PFS = progression-free survival; * Disease control rate calculated on 14 patients.

## Data Availability

The data presented in this study are available on request from the corresponding author.

## Supplementary Materials

The following supporting information can be downloaded at: https://www.mdpi.com/article/10.3390/cancers17122039/s1.
